# Thromboprophylaxis Use in Paediatric Inflammatory Bowel Disease: An International RAND Appropriateness Panel

**DOI:** 10.1093/ecco-jcc/jjac073

**Published:** 2022-05-24

**Authors:** Franco Torrente, Susanna Meade, Eric I Benchimol, Lissy de Ridder, Nicholas M Croft, Jochen Kammermeier, David R Mack, Renz C W Klomberg, Dan Turner, David C Wilson, Javier Martín-de-Carpi, Jiri Bronsky, Jorge Amil Dias, Gregor Walker, C Heleen van Ommen, Michael P Powar, Natasha Burgess, Peter M Irving, Mark A Samaan, Richard Hansen

**Affiliations:** Department of Paediatric Gastroenterology, Addenbrooke’s Hospital, Cambridge, UK; Department of Gastroenterology, Guy’s and St Thomas’ NHS Foundation Trust, London, UK; SickKids Inflammatory Bowel Disease Centre, Hospital for Sick Children; Child Health Evaluative Sciences, SickKids Research Institute; ICES; Department of Paediatrics and Institute of Health Policy, Management and Evaluation, University of Toronto, Toronto, ON, Canada; Pediatric Gastroenterology Department, Erasmus MC Sophia Children Hospital, Rotterdam, The Netherlands; Blizard Institute, Barts and the London School of Medicine, Queen Mary University of London London, UK; Paediatric Gastroenterology, Royal London Children’s Hospital, Barts Health NHS Trust London, UK; Department of Paediatric Gastroenterology, Evelina London Children’s Hospital, London, UK; CHEO IBD Centre, Children’s Hospital of Eastern Ontario Ottawa, ON, Canada; Department of Pediatrics, University of Ottawa Ottawa, ON, Canada; Pediatric Gastroenterology Department, Erasmus MC Sophia Children Hospital, Rotterdam, The Netherlands; Juliet Keidan Institute of Pediatric Gastroenterology and Nutrition, Shaare Zedek Medical Center, Hebrew University of Jerusalem, Jerusalem, Israel; Child Life and Health, University of Edinburgh Edinburgh, UK; Department of Paediatric Gastroenterology and Nutrition, Royal Hospital for Children and Young People Edinburgh, UK; Department of Pediatric Gastroenterology, Hepatology and Nutrition, Hospital Sant Joan de Déu, Barcelona, Spain; Department of Paediatrics, University Hospital Motol, Prague, Czech Republic; Department of Paediatrics, Centro Hospitalar São João, Porto, Portugal; Department of Paediatric Surgery, Royal Hospital for Children, Glasgow, UK; Pediatric Hematology Department, Erasmus MC Sophia Children Hospital, Rotterdam, The Netherlands; Cambridge Colorectal Unit, Addenbrooke’s Hospital, Cambridge, UK; Paediatric Gastroenterology, Royal London Children’s Hospital, Barts Health NHS Trust, London, UK; School of Immunology and Microbial Sciences, King’s College London London, UK; Department of Gastroenterology, Guy’s and St Thomas’ NHS Foundation Trust London, UK; Department of Gastroenterology, Guy’s and St Thomas’ NHS Foundation Trust, LondonUK; Department of Paediatric Gastroenterology, Royal Hospital for Children Glasgow, UK; Bacteria, Immunology, Nutrition, Gastroenterology and Omics Group, University of Glasgow Glasgow, UK

**Keywords:** Paediatric gastroenterology, ulcerative colitis, inflammatory bowel disease

## Abstract

**Background and Aims:**

Thromboprophylaxis use in paediatric inflammatory bowel disease [IBD] is inconsistent. Current guidelines only support treating children with acute severe colitis with risk factors. We convened an international RAND panel to explore thromboprophylaxis in paediatric IBD inpatients in the context of new evidence.

**Methods:**

We convened a geographically diverse 14-person panel of paediatric gastroenterologists alongside supporting experts. An online survey was sent before an online meeting. Panellists were asked to rate the appropriateness of thromboprophylaxis in hospitalised paediatric IBD patients via 27 scenarios of varying ages, gender, and phenotype, with and without thrombotic risk factors. Anonymised results were presented at the meeting. A second modified survey was distributed to all panellists present at the meeting. Results from the second survey constitute the RAND panel results. The validated RAND disagreement index defined disagreement when ≥ 1.

**Results:**

The combined outcome of thromboprophylaxis being considered appropriate until discharge and inappropriate to withhold was seen in 20 of 27 scenarios, including: all patients with new-onset acute severe colitis; all flares of known ulcerative colitis, irrespective of risk factors except in pre-pubescent patients with limited disease and no risk factors; and all Crohn’s patients with risk factors. Disagreement was seen in five scenarios regarding Crohn’s without risk factors, where outcomes were already uncertain.

**Conclusions:**

RAND panels are an established method to assess expert opinion in areas of limited evidence. This work therefore constitutes neither a guideline nor a consensus; however, the findings suggest a need to re-evaluate the role of thromboprophylaxis in future guidelines.

## 1. Introduction

Venous thromboembolism [VTE] is known to be a major complication of inflammatory bowel disease [IBD]. The pathophysiology of thrombosis in IBD is multifactorial and is well embraced in the famous Virchow’s triad: venous stasis, hypercoagulability, and vein damage. Each of these can perhaps best be exemplified in IBD through bed rest, inflammation, and the need for surgery.

Until recently, robust data on the incidence and outcome of VTE in children were lacking. A large Canadian population-based study^1^ including an excess of 3500 children with IBD demonstrated that the incidence of VTE episodes was 3.5 times higher than previously reported in Denmark.^2^ Not surprisingly, the incidence of VTE episodes was significantly higher in children with IBD compared with children without IBD included in the same study. The risk of thrombosis was higher within the first year after the diagnosis of IBD, suggesting that uncontrolled active inflammation could play a significant aetiological role.

As part of the PIBD-SET Quality Safety Registry initiative,^3^ data collected from around 25 000 children with IBD from 30 countries described an incidence of VTE episodes over a 4-year period nearly 14-fold higher than the pooled incidence rate of the general paediatric population. In this study, 20 VTE episodes were reported: 14 had a diagnosis of ulcerative colitis [UC]/IBD type unclassified, whereas six patients had Crohn’s disease with colonic involvement, highlighting that active colonic inflammation could be a potential risk factor for the development of VTE. In this voluntary reporting registry, which might therefore over-estimate the severity of events, cerebral sinus venous thrombosis was documented in 50% of cases and the overall mortality was 10%. In absolute terms, the overall incidence of VTE episodes in children with IBD remains very low; however, individualised outcomes from this complication can be both catastrophic and irreversible.

According to the most recent joint European Crohn’s and Colitis Organisation [ECCO] and European Society of Paediatric Gastroenterology Hepatology and Nutrition [ESPGHAN] guidelines,^4^ VTE prophylaxis with subcutaneous injections of low molecular weight heparin [LMWH] is indicated in children with acute severe colitis [ASC] in the presence of one or more additional risk factor, depending on the patient’s age group. If the above criteria were applied to the patients included in the PIBD-SETQ registry, only four out of the 20 children would have received LMWH, raising the question of whether the current approach to thromboprophylaxis is sufficient to prevent the majority of VTE cases. More recently, a UK-based RAND panel of paediatric IBD experts was convened to explore the impact of COVID-19 on the management of paediatric ASC^5^: one of the most surprising findings from this process was support for thromboprophylaxis in all paediatric patients with ASC, irrespective of detection of SARS-CoV-2 virus.

Paediatric gastroenterologists are perhaps cautious about prescribing thromboprophylaxis due to concerns about worsening IBD-related bleeding.^6^ The use of LMWH has however been shown to be safe both in adult^7^ and in paediatric^8^ inpatients with UC. In addition, a new international consensus on the prevention of venous and arterial thrombotic events in patients with IBD broadened the indications for thromboprophylaxis to any cause of a hospital admission.^9^ Interestingly, the authors do not specify any age restrictions but do identify age > 65 years as a specific minor risk factor. There is an urgent clinical need for clear and specific guidance on the use of thromboprophylaxis in children with IBD, to help minimise the incidence of VTE in at-risk individuals.^10^

In consideration of the above, we convened a RAND panel focusing on specific scenarios of children admitted to hospital for an exacerbation of their IBD, to support the development of specific recommendations on the appropriateness of thromboprophylaxis in this patient group.

## 2. Materials and Methods

The RAND/UCLA methodology originates from the military and was further developed in the clinical arena by the University of California, Los Angeles [UCLA]. The aim is to provide clarity on the appropriateness of interventions or treatments in specific clinical scenarios.^11^ It is, therefore, particularly useful where limited evidence exists. It is a validated method that follows a Delphi model and combines expert opinion with the best available evidence. Unlike a Delphi model, however, RAND methodology does not seek to force consensus and instead describes and reports agreement and disagreement as fundamental results of the process.

We convened a geographically –diverse 14-person panel of paediatric gastroenterologists [Supplementary Table 1]. A literature search was performed [Supplementary Table 2] and disseminated to all panellists. An online survey was created, iteratively improved, and subsequently sent to all panellists to complete ahead of an online panel meeting scheduled for October 2021. According to RAND methodology, a panel of 12–15 members is considered the optimum number to allow full panel discussion at the meeting.^11^ Panellists were asked to rate the appropriateness of prescribing thromboprophylaxis in hospitalised patients with IBD in specific clinical scenarios. Three broad categories of IBD were explored: new-onset acute severe colitis, known Crohn’s disease with a severe flare requiring hospitalisation, and known ulcerative colitis with a severe flare requiring hospitalisation. The specific scenarios and various assumptions are outlined in full within the survey provided to panellists [Supplementary File 1]. Phenotypes were described by Paris criteria.^12^ Appropriateness is a measure of harm versus benefit of a given intervention in a specific clinical circumstance [where 1–3 is inappropriate, 4–6 is uncertain, and 7–9 is appropriate]. Results were anonymised and presented at the online meeting. Present at the meeting were 12 expert panellists [two members were unexpectedly unable to attend], four experts (a paediatric and adult colorectal surgeon [GW and MP], a paediatric haematologist [HvO], a paediatric clinical nurse specialist [NB]) and two moderators [MAS, SM]. The aim of the meeting was to ensure a common understanding of the questions posed and to focus discussion on areas of disagreement. Differing from Delphi methodology, there was no attempt to force consensus. The experts did not vote but provided their opinion, when relevant, to aid decision making. Several assumptions were made at the outset to add clarity to the statements. Based on the literature review and expert discussion, the panellists also agreed on 13 risk factors for thrombosis relevant to paediatric IBD [Box 1]. A second modified survey comprising 82 statements was approved by, and distributed to, all panellists present at the meeting [Supplementary File 1]. Results from the second survey constitute the RAND panel results.

**Box 1 Thrombotic risk factors extrapolated from the literature and agreed by the panellists**

**Thrombotic risk factors in paediatric inflammatory bowel disease**

Severe disease

Surgery

Smoking

Oral contraceptive pill

Complete immobilisation

Central venous catheter or peripherally inserted central catheter

Obesity

Concurrent significant infection

Known thrombotic disorder

Previous thrombosis

Family history of thrombosis

Systemic steroids

Parenteral nutrition.

### 2.1. Analysis

A median appropriateness score was calculated for each statement where a score of < 3.5 was deemed inappropriate, ≧3.5 to < 6.5 uncertain, and ≧6.5 to 9.0 appropriate. The disagreement index [DI] is a validated score and, as per methodology, a score of ≧1 was used to define disagreement. If disagreement should be present, the outcome would be rated as uncertain, irrespective of the median appropriateness score.


$$\begin{array}{l} DI=\frac{70\;\%\;ile-30\;\%\;ile}{2.35+\left(1.5\times abs\left(5-\frac{70\;\%\;ile+30\;\%\;ile}{2}\right)\right)} \end{array}$$

## 3. Results

All 12 panellists voted on each of the 82 statements. Twenty statements were rated as inappropriate, 38 statements as uncertain, and 24 statements as appropriate. Disagreement was reached for five statements, all of which had an initial rating of uncertain and pertained to patients flaring with Crohn’s disease. A detailed breakdown of the statements with median score, disagreement index, standard deviation, interpercentile range, and final RAND panel outcome is shown in Supplementary Table 3.

Table 1 shows the results for patients admitted with new-onset ASC. It was considered both appropriate to offer thromboprophylaxis until discharge and inappropriate to omit this therapy in all patients, irrespective of pubertal status or sex. Also, irrespective of pubertal status and sex, it was considered uncertain as to whether this should be continued after discharge until clinical remission was achieved.

**Table 1.**
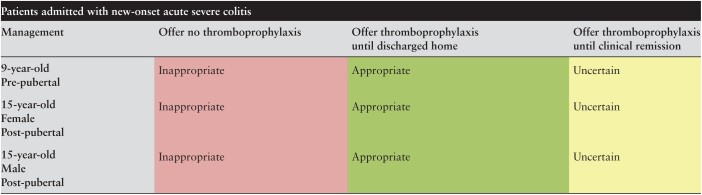
Appropriateness of thromboprophylaxis in paediatric patients admitted with first presentation of acute severe colitis.

Green is considered appropriate, yellow uncertain, and red inappropriate.

Table 2 shows the results for patients admitted with an acute flare of severe UC. In all patients, irrespective of disease extent, sex, pubertal status, and the presence or absence of additional VTE risk factors, it was considered appropriate to offer thromboprophylaxis until discharge. Greater phenotypic disease extent such as extensive colitis and pancolitis, with the presence of an additional VTE risk factor, resulted in higher median scores [E3/4 disease with additional risk factors median scores 8.5–9.0] whereas the converse was true for patients with limited disease and no additional risk factor [median scores 7]. The prescription of LMWH until clinical remission [essentially, continuation of LMWH after discharge until remission was achieved] was considered uncertain in all patients. It was considered inappropriate to omit thromboprophylaxis in all patients except pre-pubescent patients with limited disease extent and no additional risk factors where the outcome was deemed uncertain.

**Table 2.**
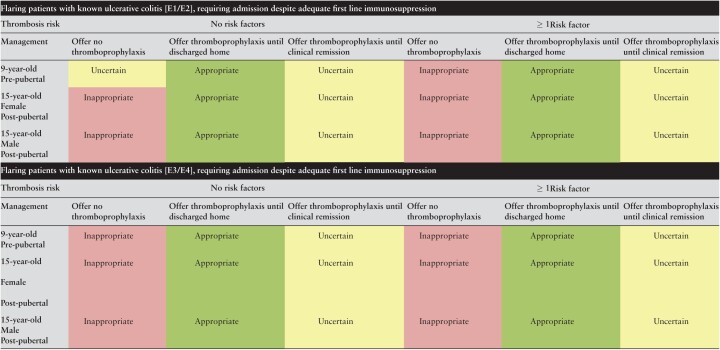
Appropriateness of thromboprophylaxis in paediatric patients admitted with flare of known ulcerative colitis.

Green is considered appropriate, yellow uncertain, and red inappropriate.

Table 3 shows the results for patients with severe Crohn’s disease requiring admission for an acute flare. Irrespective of pubertal status or sex, it was considered uncertain as to whether patients with limited ileal disease [L1B1 phenotype] with no additional risk factors for VTE should be offered thromboprophylaxis during admission or after discharge. Not only was there uncertainty, but three of five statements where disagreement was reached occurred in this category, highlighting the range of opinion. There was more clarity however, in patients with limited ileal disease where an additional risk factor for VTE was present, again irrespective of sex or pubertal status. In those cases, it was considered inappropriate to omit thromboprophylaxis and appropriate to provide LMWH until discharge. The prescription of LMWH until clinical remission in these patients was considered uncertain. Conversely, where patients were admitted with an acute flare of Crohn’s disease with colonic involvement [L2- colonic or L3- ileocolonic distribution], it was considered appropriate to offer thromboprophylaxis to all patients until discharge. Age and pubertal status had no effect on the median scores, whereas the presence of an additional VTE risk factor increased the median scores from 7 to 8. Omission of prophylaxis was considered uncertain in patients with colonic Crohn’s and no risk factors [with disagreement regarding pre-pubescent and male post-pubescent patients]. Among children with an additional risk factor for VTE, however, it was considered inappropriate to omit thromboprophylaxis. In all cases it was uncertain as to whether thromboprophylaxis should be continued after discharge.

**Table 3.**
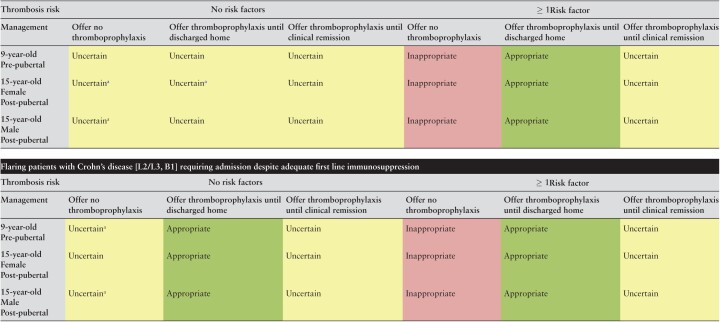
Appropriateness of thromboprophylaxis in paediatric patients admitted with flare of known Crohn’s disease; flaring patients with Crohn’s disease [L1B1] requiring admission despite adequate first-line immunosuppression.

[Green is considered appropriate, yellow uncertain. and red inappropriate.

Denotes disagreement index ≥ 1].

**Figure 1:**
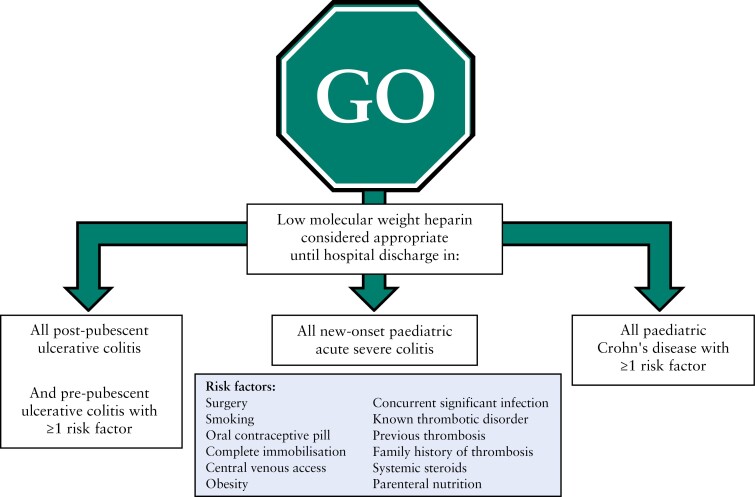
Summary diagram of hospitalised paediatric patients with severe inflammatory bowel disease where prophylactic treatment with heparin considered by RAND panel as both appropriate to administer and inappropriate to withhold.

The results of Tables 1–3 are summarised in Figure 1 alongside the risk factors from Box 1, to help facilitate consideration of thromboprophylaxis in day-to-day clinical care.

Clinical practice with regards to the safety of proceeding with endoscopy and biopsy on prophylactic heparin varies geographically and between institutions. We therefore asked panellists whether it was appropriate to proceed with endoscopy and biopsy without interrupting heparin. The outcome was uncertain.

In the pre-panel survey, we had included a further section on hospitalised patients with severe UC or CD requiring inpatient surgery. Despite the presence of two surgical experts, the panel could not agree that it was the role of the gastroenterological team to make these decisions and therefore these statements were removed.

## 4. Discussion

The need for this RAND panel was realised by the confluence of three distinct contributions to the literature: first, the emergence of new Canadian paediatric population-based epidemiology data increasing the perceived size of the problem 3-fold^1^; second, the description of a VTE cohort including 50% of cases with cerebral thrombosis and a 10% overall mortality^3^; and third, the emergence of recommendations from a previous RAND panel to offer LMWH to all children with ASC during the COVID-19 pandemic, irrespective of whether there was infection with SARS-CoV-2.^5^ Considering these in the context of guidelines supporting use of LMWH only in patients with risk factors,^4^ it was clear that a targeted update was required; however, this remains an academic area with a paucity of supporting literature. A RAND panel was therefore considered to be the ideal methodology, given its specific function in this niche. It is, however, important in this context to reiterate that a RAND panel is not a guideline development process. It is hoped that this work will stimulate further research in the field and support future guidelines in paediatric IBD by bringing forth specific detailed consideration in a challenging subject area, potentially helping facilitate a change in practice. Since the RAND panel did not undergo a Delphi consensus, the reported results are not the opinion of all authors of this work. No attempt is made to seek consensus during the RAND process, with panellists free to vote however they wish on any clinical scenario. Ultimately three categorical options are available, with three levels of strength each, namely: appropriate, uncertain, or inappropriate. Disagreement is defined mathematically where the panel are not aligned, and in such cases the outcome is automatically considered uncertain.

Figure 1 offers a summary of the outcomes of this RAND panel for consideration in clinical practice. In short, three groups of children admitted to hospital for severe IBD can be defined in whom LMWH would be reasonable to offer until discharge. Each of these is defined by the combined outcome of LMWH being considered appropriate and its omission inappropriate. These three groups are: all children with new-onset ASC; all post-pubertal children with UC and pre-pubescent children with UC and more than one risk factor; and all children with Crohn’s disease and more than one risk factor. It is important to note the limitations of this work in providing evidence on a case-by-case basis, however. In order to generate a workable survey, broad categories were used to explore themes. For instance, pre-pubertal children were represented by a 9-year-old and post-pubertal children were represented by a 15-year-old. Neither captures the full range of possibilities, and the youngest children in particular are perhaps poorest served by this conceit, despite increasing incidence of very early-onset IBD.^13,14^ Similarly, the Paris modification of the Montreal classification of IBD^12^ was used as shorthand for disease phenotype with amalgamation, for example, of E1 and E2 UC [proctitis and left-sided colitis, respectively] being undertaken for convenience. We recognise that such amalgamations include a huge potential phenotypic range in practice, and that E1 cases are also perhaps unlikely to end up hospitalised for their disease, but we felt that exploration of distinct disease phenotypes was a worthwhile undertaking to help explore how opinions might change among panellists. These limitations do reduce generalisability of our findings to an individual patient level, but the multiplication effect of offering distinct options per clinical scenario meant we had to make a pragmatic choice to reflect some of the gradations seen between patients but not every distinct feature. We believe our compromises still allowed us to describe recognisable and clinically applicable scenarios from everyday practice.

Although recent data suggest that VTE incidence is higher than previously reported,^1^ the absolute risk of venous thromboembolism in children with inflammatory bowel disease remains very low. Conducting a meaningful randomised study on thromboprophylaxis in this group of children would therefore prove to be impractical, if not impossible, and likely to be considered unethical if using placebo; not surprisingly, the current recommendations for thromboprophylaxis^4^ are based on very limited evidence. In contrast, our RAND process concluded that the indications for thromboprophylaxis, rather than being limited to cases of ASC with additional risk factors, could potentially be extended to a much larger group of children with IBD in need of hospitalisation. We acknowledge the current lack of evidence that this intervention would necessarily prevent thrombotic events in the PIBD setting, but note the unequivocal evidence base for LMWH thromboprophylaxis in reducing VTE episodes from adult literature.^15,16^ Conversely, among the 20 patients who developed VTE in the international safety registry,^3^ only four of the cases would have received LMWH according to existing guidelines. It could be argued that the number of children needing treatment with LMWH to prevent one episode of VTE is difficult to calculate and could be high. Debating the usefulness of this intervention, however, three further aspects need to be taken into consideration: first, and most importantly, the potential severity of VTE. Although there might be a recall bias favouring the publication of cases with the worst outcomes, there is no doubt that complications can be severe, debilitating, and, in some circumstances, catastrophic. Second, the administration of LMWH in children has been shown to be safe. Earlier data from a meta-analysis on the use of heparin in patients with UC^7^ have recently been confirmed by a retrospective cohort study of 218 paediatric inpatients with active UC, showing that there was no difference in haemoglobin levels or need for blood transfusions in children hospitalised for ASC whether or not they received enoxaparin for thromboembolism prophylaxis.^8^ Finally, it is important that paediatricians carefully consider the pain and anxiety of frequent injections in a population who are already under significant medical and emotional stress. This latter point may be amplified by patient-specific factors in some cases and is certainly worth discussing with individual families.

There is no evidence to support a specific duration of LMWH therapy in this setting. Recently published adult data^17^ indicate that urgent surgery for UC was associated with an increased risk of VTE and that the risk was greatest 2 weeks after discharge. The authors therefore concluded that for this group of patients, health care providers should consider an extended period of thromboprophylaxis after hospital discharge. We sought to explore duration by offering ‘… until discharged home’ against ‘… until clinical remission’. The latter choice was always considered uncertain by our panel. This is perhaps something to be returned to if LMWH usage increases in the PIBD inpatient population, as changes in VTE incidence and timing could be explored against changes in practice and this may help ascertain the most appropriate duration. For now, LMHW is considered appropriate for PIBD inpatient care only.

The first set of clinical questions proposed to the RAND panellists included patients admitted to hospital to undergo surgery, and we ensured that the non-voting group of experts had representation from both adult and paediatric surgery to facilitate this. However, the discussion during the virtual meeting highlighted the difference of approaches and practices between different centres and countries, making any generalisation very difficult. Due to this, it was concluded that it was not possible to make any recommendation involving this group of children and the decision was made to drop these scenarios from the final questionnaire. Importantly, however, it was agreed that surgery by itself represented a risk factor for thrombotic events, and one interpretation of our RAND results would support the use of LMWH in surgical PIBD patients who inherently have a risk factor at baseline. Practically, however, the consideration of LMHW use in PIBD patients undergoing surgery requires further targeted work and broader engagement from our surgical colleagues, particularly regarding both timing and risk of bleeding.

This RAND panel has given new and focused consideration to an increasingly important but contentious subject in PIBD practice, namely the use of LMWH thromboprophylaxis in inpatient care. Though not a guideline, this work provides targeted and clinically useful reflection on when LMWH should be considered appropriate in PIBD care. It is hoped that this work will help reduce the incidence, morbidity, and mortality of a potentially devastating but avoidable complication in our patient population.

All data are incorporated into the article and its online Supplementary material.

## Supplementary Material

jjac073_suppl_Supplementary_File_1

jjac073_suppl_Supplementary_Tables

## Data Availability

All data analysed for this paper are either presented within the main manuscript or within the Supplementary Files.
