# Supplementary material for: Thromboprophylaxis Use in Paediatric Inflammatory Bowel Disease: An International RAND Appropriateness Panel
Source: J Crohns Colitis. 2022 May 24;16(10):1609–16. doi: 10.1093/ecco-jcc/jjac073 (PMC9624289; doi:10.1093/ecco-jcc/jjac073)
Supplement: jjac073_suppl_Supplementary_File_1 [file jjac073_suppl_supplementary_file_1.pdf]

Thromboprophylaxis in paediatric IBD - A RAND appropriateness panel. Post-meeting questionnaire

Thromboprophylaxis in paediatric IBD - POST PANEL MEETING QUESTIONNAIRE

Thank you in advance for completing this survey.

Please complete the survey within the next week if at all possible.

* 1. Please type your name

Thromboprophylaxis in paediatric IBD - A RAND appropriateness panel. Post-meeting questionnaire

INTRODUCTION

Dear Colleagues,

The British Society of Paediatric Gastroenterology, Hepatology and Nutrition (BSPGHAN) IBD Working Group recently published a paper derived from a RAND panel convened to explore the impact of COVID-19 on management of acute severe colitis. (1) One of the more surprising findings from this process was support for thromboprophylaxis in all patients with acute severe colitis, irrespective of Covid status. Distinctly from this, an international prospective cohort study recently reported on the incidence of venous thromboembolism in paediatric inflammatory bowel disease patients and proposed new recommendations about thromboprophylaxis in our patient population. (2)
We therefore thought this a good time to convene a distinct RAND panel to explore approaches to thrombosis in inpatient paediatric IBD management and, in doing so, to offer recommendations to support practice in this area in the absence of high-quality evidence

The RAND methodology we are using combines expert opinion with the best available evidence. There is no "right answer" to any question, but you will be asked to grade how appropriate specific interventions / actions are using a scale from 1-9.

1-3 = inappropriate (intervention causes more harm than benefit)

4-6 = uncertain (panellist cannot make a judgement with the available evidence or harm=benefit)

7-9 = appropriate (intervention is more beneficial than it is harmful)

We accept there is likely to be limited evidence to support your ratings. This is the reason to use the RAND methodology as it is designed to guide how people should act in situations where the evidence is limited. As you go through the survey, feel free to refer to the Literature Review, or any other literature, as you see fit. There will be some scenarios where you might think "it depends," but please give your best possible answer.

Although scenarios may seem monotonous as you go through, please pay attention to the instructions at the top of the screen as variables are altered with each screen to see how different factors influence your decision making. Your ratings are confidential, and will remain so unless you choose to reveal your ratings to the other panelists at the virtual meeting. You will be asked to enter your name so that respondents can be tracked. At the end of the survey, you will have the opportunity to suggest any additional questions or scenarios which will be discussed at the virtual meeting.

At the virtual meeting, you should record your scores again on a paper copy of the survey (we will provide a PDF for you to print) in light of any discussion that takes place after which you will be sent another link to the survey to input your new scores (whether they have changed or not).

Please note the following assumptions/instructions that apply to all scenarios:

1. Where an IBD diagnosis or phenotype is described, this is based on a complete assessment in keeping with the revised Porto criteria (3), with phenotyping in keeping with Paris criteria (4).

2. Where disease activity is discussed as remission/mild/moderate/severe, this is based on weighted paediatric Crohn's disease activity index (5) or paediatric ulcerative colitis activity index (6) scores respectively.

3. Medical therapy, where relevant, is briefly described in each scenario but has been chosen and optimised specific to the scenario in question and with respect to the time allowed to do so. You are not being asked to choose a medical therapy, so when medical therapy is discussed in the stem, this is to allow us to capture thinking on thromboprophylaxis across the spectrum of options. Please answer all stems without consideration of whether or not you would approach the scenario itself differently.

4. All patients included in the scenarios do not present any additional non-IBD risk factor for a thrombotic complication (central venous catheter, total parental nutrition, etc.)

5. Treatment initiated during the admission in question, in particular steroids, counts as a risk factor within those stated in each scenario. Those that state "no associated risk factors" therefore naturally define a patient where steroids are not part of the treatment course at the point of heparin introduction. Use of steroids in treating the scenario naturally dictates a patient with "one or more associated risk factors". For this reason, "no associated risk factors" scenarios are not included for newly presenting acute severe colitis patients as steroid treatment is an integral part of their medical therapy.

6. Surgery also counts as a risk factor and so the "one or more associated risk factors" scenarios can be presumed to apply to patients with the inflammatory burden described who are admitted requiring surgery. Agreement on use of heparin in the perioperative period obviously requires further discussion and agreement with surgical colleagues.

7. When recommending thromboprophylaxis, this relates to a prescription of low molecular weight heparin (LMWH) given daily at a dose in keeping with prophylaxis for thrombotic episodes e.g. Enoxaparin 500micrograms/kg subcutaneously twice daily; maximum 40mg per day (taken from BNF-C 2021).

Please also note that the RAND methodology instructs respondents to make decisions WITHOUT taking into account local availability of treatments or cost.

References:

1. Hansen R et al Gut 2021 doi: 10.1136/gutjnl-2020-322449.

2. Aardoom MA, Klomberg RCW, et al. Manuscript submitted.

3. Levine A, et al. JPGN 2014; 58(6): 795-806.

4. Levine A, et al. Inflamm Bowel Dis 2011; 17(6): 1314-1321.

5. Turner D, et al. Inflamm Bowel Dis 2012; 18(1): 55-62.

6. Turner D, et al. Gastroenterology 2007; 133(2): 423-432.

Adult and paediatric IBD guideline recommendations and prospective registry data regarding VTE

Please remind yourself of the current guidance for thromboprophylaxis from both adult and paediatric practice. In addition, recent data from a prospective IBD registry. (Table adapted from Aardoom MA, Klomberg RCW, et al.)

Age Range	Guideline and/or consensus statements	Recommendations
**Adult**	**ACG Guideline for adult UC patients**<br><br>Rubin DT, et al. ACG clinical guideline: Ulcerative colitis in adults. Am J Gastroenterol 2019; 114(3): 384-413.	VTE prophylaxis with LMWH for hospitalized patients with acute severe colitis
**Adult**	**BSG Guideline for IBD patients**<br><br>Lamb CA, et al. British Society of Gastroenterology consensus guidelines on the management of inflammatory bowel disease in adults. Gut 2019; 68(Supp 3): S1-S106.	VTE prophylaxis with LMWH for hospitalized patients with acute severe colitis
**Adult**	**ECCO Guideline for extra-intestinal manifestations in adult IBD patients**<br><br>Harbord M, et al. The first European evidence-based consensus on extra-intestinal manifestations in inflammatory bowel disease. J Crohns Colitis 2016; 10(3): 239-254.	VTE prophylaxis is recommended for all hospitalized IBD patients and should be considered following discharge from hospital and after recent surgery, and in outpatients with active disease
**Adult**	**ACG Guideline for adult CD patients**<br><br>Lichtenstein GR, et al. ACG clinical guideline: Management of Crohn's disease in adults. Am J Gastroenterol 2018; 113(4): 481-517.	No recommendations
**Paediatric**	**ESPGHAN/ECCO Guideline for paediatric UC patients (part 2) – Acute Severe Colitis**<br><br>Turner D, et al. Management of paediatric ulcerative colitis, part 2: Acute severe colitis... JPGN 2018; 67(2): 292-310.	VTE prophylaxis with LMWH for paediatric patients with acute severe colitis when 1 or more risk factors are present.<br><br>Risk factors include: smoking, oral contraceptives, complete immobilization, CVCs (including PICC line), obesity, concurrent significant infection, known thrombotic disorder, previous VTE and family history of VTE.
**Paediatric**	**ESPGHAN/ECCO Guideline for pediatric CD patients (update)**<br><br>van Rheenen PF, et al. The medical management of paediatric Crohn's disease: an ECCO-ESPGHAN Guideline Update. J Crohns Colitis 2021; 15(2): 171-194.	No recommendations
**Paediatric**	**ESPGHAN/ECCO Guideline for paediatric UC patients (part 1) – ambulatory care**<br><br>Turner D, et al. Management of paediatric ulcerative colitis, part 1: Ambulatory care... JPGN 2018; 67(2): 257-91.	No recommendations
**All ages**	**CAG Consensus statements for VTE prevention in IBD patients**<br><br>Nguyen GC, et al. Consensus statements on the risk, prevention, and treatment of venous thromboembolism in inflammatory bowel disease: Canadian Association of Gastroenterology. Gastroenterology 2014; 146(3): 835-848.	1) VTE prophylaxis with LMWH, low-dose UH or fondaparinux for all hospitalized adult IBD patients.<br>2) No VTE prophylaxis for children with IBD-related hospitalization without a previous VTE

Age range	Prospective data	Recommendations
**Paediatric**	**Prospective international cohort study on the incidence of VTE in paediatric IBD**<br><br>Aardoom MA, Klomberg RCW, Kemos P, et al. The Incidence and Characteristics of Venous Thromboembolisms in Paediatric-Onset Inflammatory Bowel Disease: A Prospective International Cohort Study Based on the PIBD-SETQuality Safety Registry. Journal of Crohn's and Colitis 2021.	Consider thromboprophylaxis in hospitalized children with moderate-to-severe active Crohn's disease with at least one additional risk factor for VTE.<br>Non-IBD risk factors defined as:<br>· Steroid usage, immobility, central venous catheter, parenteral nutrition, surgery<br><br>Considering the increased risk of VTE and the possible poor outcome advise to consider thromboprophylaxis for all hospitalized patients with ASC, regardless of age and the presence of additional risk factors.

Previous page: Overview of international adult and paediatric inflammatory bowel disease guidelines and the reported recommendations regarding thromboembolic prophylaxis. ACG, American College of Gastroenterology; BSG, British Society of Gastroenterology; CAG, Canadian Association of Gastroenterology; JCC, Journal of Crohn's and Colitis; JPGN, Journal of Paediatric Gastroenterology and Nutrition; CD, Crohn's disease; CVC, central venous catheter; ECCO, European Crohn's and Colitis Organisation; ESPGHAN, European Society for Paediatric Gastroenterology Hepatology and Nutrition; IBD, inflammatory bowel disease; LMWH, low molecular weight heparin; PICC, peripherally inserted central catheter; UC, ulcerative colitis; UH, unfractionated heparin; VTE, venous thromboembolism; PIBD-SETQuality, paediatric inflammatory bowel disease network for safety, efficacy, treatment and quality improvement of care.

List of thrombotic risk factors extrapolated from the literature search and agreed by the panellists:

Severe disease
Surgery
Smoking
Oral contraceptive pill
Complete immobilisation
Central venous catheters or PICC lines
Obesity
Concurrent significant infection
Known thrombotic disorder
Previous VTE
Family history of VTE
Systemic steroids
Parenteral nutrition

Please consider these previous RAND panel recommendations on thromboprophylaxis in paediatric acute severe colitis (taken from Hansen R, et al. Gut 2021 doi: 10.1136/gutjnl-2020-322449):

Table 2 Appropriateness of treatment options in acute severe colitis in the context of the COVID-19 pandemic: first-line medical therapy

	First-line medical therapy					
Negative SARS-CoV-2 swab without signs of COVID-19 infection	IP IV corticosteroids	Poorly bioavailable steroids*	IFX alone	Tacrolimus±corticosteroids	MADoV alone	Discussion with COVID-19 specialist†
	OP IV corticosteroids	IV corticosteroids+IFX	Ciclosporin±corticosteroids	MADoV as adjunctive therapy	Thromboprophylaxis	
Positive SARS-CoV-2 swab without signs of COVID-19 infection	IP IV steroids	Poorly bioavailable steroids*	IFX alone	Tacrolimus±corticosteroids	MADoV alone	Discussion with COVID-19 specialist†
	OP IV corticosteroids	IV corticosteroids+IFX	Ciclosporin±corticosteroids	MADoV as adjunctive therapy	Thromboprophylaxis	
Positive SARS-CoV-2 swab with signs of COVID-19 infection	IP IV corticosteroids	Poorly bioavailable steroids*	IFX alone	Tacrolimus±corticosteroids	MADoV alone	Discussion with COVID-19 specialist†
	OP IV corticosteroids	IV corticosteroids+IFX	Ciclosporin±corticosteroids	MADoV as adjunctive therapy	Thromboprophylaxis	

Green is considered appropriate, yellow uncertain and red inappropriate.
*Budesonide MMX/beclometasone.
†Discussion with appropriate COVID-19 specialist as per local availability.
IFX, infliximab; IP, inpatient; IV, intravenous; MADoV, metronidazole, amoxicillin, doxycycline, vancomycin or equivalent; methylprednisolone, corticosteroids; MMX, multimatrix; OP, outpatient.

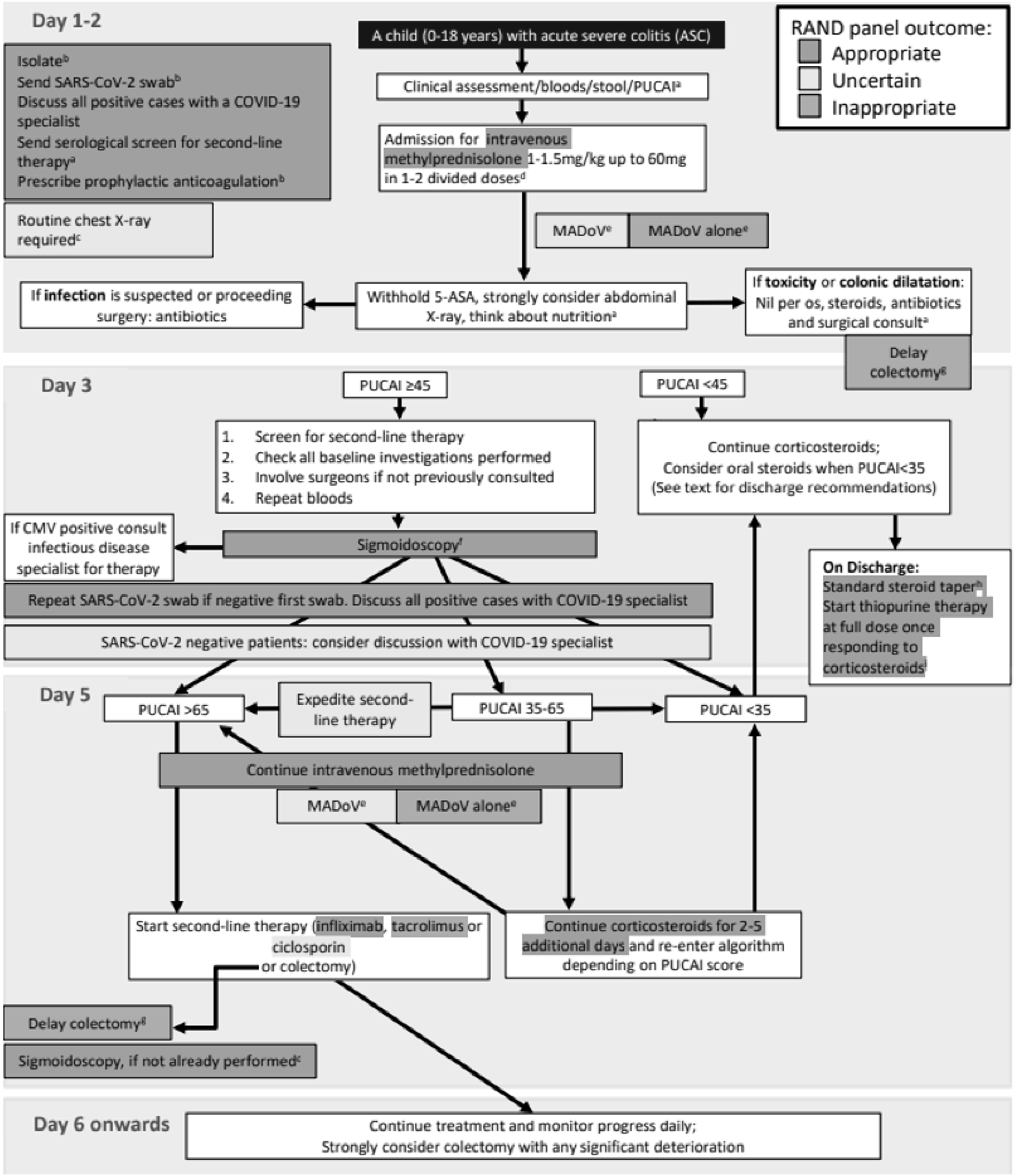

Day 1-2
A child (0-18 years) with acute severe colitis (ASC)
RAND panel outcome:
Appropriate
Uncertain
Inappropriate
Isolate[b]
Send SARS-CoV-2 swab[b]
Discuss all positive cases with a COVID-19 specialist
Send serological screen for second-line therapy[a]
Prescribe prophylactic anticoagulation[b]
Clinical assessment/bloods/stool/PUCAI[a]
Admission for intravenous methylprednisolone 1-1.5mg/kg up to 60mg in 1-2 divided doses[d]
Routine chest X-ray required[c]
MADoV[e]
MADoV alone[e]
If infection is suspected or proceeding surgery: antibiotics
Withhold 5-ASA, strongly consider abdominal X-ray, think about nutrition[a]
If toxicity or colonic dilatation: Nil per os, steroids, antibiotics and surgical consult[a]
Delay colectomy[g]
Day 3
PUCAI ≥45
PUCAI <45
1. Screen for second-line therapy
2. Check all baseline investigations performed
3. Involve surgeons if not previously consulted
4. Repeat bloods
Continue corticosteroids;
Consider oral steroids when PUCAI<35
(See text for discharge recommendations)
If CMV positive consult infectious disease specialist for therapy
Sigmoidoscopy[f]
On Discharge:
Standard steroid taper[h]
Start thiopurine therapy at full dose once responding to corticosteroids[i]
Repeat SARS-CoV-2 swab if negative first swab. Discuss all positive cases with COVID-19 specialist
SARS-CoV-2 negative patients: consider discussion with COVID-19 specialist
Day 5
PUCAI >65
Expedite second-line therapy
PUCAI 35-65
PUCAI <35
Continue intravenous methylprednisolone
MADoV[e]
MADoV alone[e]
Start second-line therapy (infliximab, tacrolimus or ciclosporin or colectomy)
Continue corticosteroids for 2-5 additional days and re-enter algorithm depending on PUCAI score
Delay colectomy[g]
Sigmoidoscopy, if not already performed[c]
Day 6 onwards
Continue treatment and monitor progress daily;
Strongly consider colectomy with any significant deterioration

Chapter 1: New-onset acute severe colitis

A) In a 9 year old (pre-pubescent) patient with newly diagnosed acute severe colitis with one or more associated risk factors for venous thrombosis after limited sigmoidoscopy

(1-3 = inappropriate, 4-6 = uncertain, 7-9 = appropriate)

* 1. Offer no thromboprophylaxis

* 2. Offer thromboprophylaxis until discharged home

* 3. Offer thromboprophylaxis until in clinical remission

Thromboprophylaxis in paediatric IBD - A RAND appropriateness panel. Post-meeting questionnaire

Chapter 1: New-onset acute severe colitis

B) In a 15 year old (post-pubescent) female patient with newly diagnosed acute severe colitis with one or more associated risk factors for venous thrombosis after limited sigmoidoscopy

(1-3 = inappropriate, 4-6 = uncertain, 7-9 = appropriate)

* 1. Offer no thromboprophylaxis

* 2. Offer thromboprophylaxis until discharged home

* 3. Offer thromboprophylaxis until in clinical remission

Thromboprophylaxis in paediatric IBD - A RAND appropriateness panel. Post-meeting questionnaire

Chapter 1: New-onset acute severe colitis

C) In a 15 year old (post-pubescent) male patient with newly diagnosed acute severe colitis with one or more associated risk factors for venous thrombosis after limited sigmoidoscopy

(1-3 = inappropriate, 4-6 = uncertain, 7-9 = appropriate)

* 1. Offer no thromboprophylaxis

* 2. Offer thromboprophylaxis until discharged home

* 3. Offer thromboprophylaxis until in clinical remission

Thromboprophylaxis in paediatric IBD - A RAND appropriateness panel. Post-meeting questionnaire

Chapter 2a: Flaring patients with Crohn's disease requiring hospital admission

A) In a 9 year old (pre-pubescent) patient with known Crohn’s disease with severe active disease in an “ileal” distribution (Paris L1, B1) despite adequate first line immunosuppression and no associated risk factors for venous thrombosis

(1-3 = inappropriate, 4-6 = uncertain, 7-9 = appropriate)

* 1. Offer no thromboprophylaxis

* 2. Offer thromboprophylaxis until discharged home

* 3. Offer thromboprophylaxis until in clinical remission

Chapter 2a: Flaring patients with Crohn's disease requiring hospital admission

B) In a 9 year old (pre-pubescent) patient with known Crohn’s disease with severe active disease in an “ileal” distribution (Paris L1, B1) despite adequate first line immunosuppression and one or more associated risk factors for venous thrombosis

(1-3 = inappropriate, 4-6 = uncertain, 7-9 = appropriate)

* 1. Offer no thromboprophylaxis

* 2. Offer thromboprophylaxis until discharged home

* 3. Offer thromboprophylaxis until in clinical remission

Thromboprophylaxis in paediatric IBD - A RAND appropriateness panel. Post-meeting questionnaire

Chapter 2a: Flaring patients with Crohn's disease requiring hospital admission

C) In a 15 year old (post-pubescent) female patient with known Crohn’s disease with severe active disease in an “ileal” distribution (Paris L1, B1) despite adequate first line immunosuppression and no associated risk factors for venous thrombosis.

(1-3 = inappropriate, 4-6 = uncertain, 7-9 = appropriate)

* 1. Offer no thromboprophylaxis

* 2. Offer thromboprophylaxis until discharged home

* 3. Offer thromboprophylaxis until in clinical remission

Chapter 2a: Flaring patients with Crohn's disease requiring hospital admission

D) In a 15 year old (post-pubescent) female patient with known Crohn's disease with severe active disease in an "ileal" distribution (Paris L1, B1) despite adequate first line immunosuppression and one or more associated risk factors for venous thrombosis.

(1-3 = inappropriate, 4-6 = uncertain, 7-9 = appropriate)

* 1. Offer no thromboprophylaxis

* 2. Offer thromboprophylaxis until discharged home

* 3. Offer thromboprophylaxis until in clinical remission

Thromboprophylaxis in paediatric IBD - A RAND appropriateness panel. Post-meeting questionnaire

Chapter 2a: Flaring patients with Crohn's disease requiring hospital admission

E) In a 15 year old (post-pubescent) male patient with known Crohn's disease with severe active disease in an "ileal" distribution (Paris L1, B1) despite adequate first line immunosuppression and no associated risk factors for venous thrombosis.

(1-3 = inappropriate, 4-6 = uncertain, 7-9 = appropriate)

* 1. Offer no thromboprophylaxis

* 2. Offer thromboprophylaxis until discharged home

* 3. Offer thromboprophylaxis until in clinical remission

Chapter 2a: Flaring patients with Crohn's disease requiring hospital admission

F) In a 15 year old (post-pubescent) male patient with known Crohn’s disease with severe active disease in an “ileal” distribution (Paris L1, B1) despite adequate first line immunosuppression and one or more associated risk factors for venous thrombosis.

(1-3 = inappropriate, 4-6 = uncertain, 7-9 = appropriate)

* 1. Offer no thromboprophylaxis

* 2. Offer thromboprophylaxis until discharged home

* 3. Offer thromboprophylaxis until in clinical remission

Thromboprophylaxis in paediatric IBD - A RAND appropriateness panel. Post-meeting questionnaire

Chapter 2a: Flaring patients with Crohn's disease requiring hospital admission

G) In a 9 year old (pre-pubescent) patient with known Crohn’s disease with severe active disease in a “colonic/ileocolonic” distribution (Paris L2 or L3, B1) despite adequate first line immunosuppression and no associated risk factors for venous thrombosis.

(1-3 = inappropriate, 4-6 = uncertain, 7-9 = appropriate)

* 1. Offer no thromboprophylaxis

* 2. Offer thromboprophylaxis until discharged home

* 3. Offer thromboprophylaxis until in clinical remission

Chapter 2a: Flaring patients with Crohn's disease requiring hospital admission

H) In a 9 year old (pre-pubescent) patient with known Crohn’s disease with severe active disease in a “colonic/ileocolonic” distribution (Paris L2 or L3, B1) despite adequate first line immunosuppression and one or more associated risk factors for venous thrombosis.

(1-3 = inappropriate, 4-6 = uncertain, 7-9 = appropriate)

* 1. Offer no thromboprophylaxis

* 2. Offer thromboprophylaxis until discharged home

* 3. Offer thromboprophylaxis until in clinical remission

Thromboprophylaxis in paediatric IBD - A RAND appropriateness panel. Post-meeting questionnaire

Chapter 2a: Flaring patients with Crohn's disease requiring hospital admission

I) In a 15 year old (post-pubescent) female patient with known Crohn’s disease with severe active disease in a “colonic/Ileocolonic” distribution (Paris L2 or L3, B1) despite adequate first line immunosuppression and no associated risk factors for venous thrombosis.

(1-3 = inappropriate, 4-6 = uncertain, 7-9 = appropriate)

* 1. Offer no thromboprophylaxis

* 2. Offer thromboprophylaxis until discharged home

* 3. Offer thromboprophylaxis until in clinical remission

Thromboprophylaxis in paediatric IBD - A RAND appropriateness panel. Post-meeting questionnaire

Chapter 2a: Flaring patients with Crohn's disease requiring hospital admission

J) In a 15 year old (post-pubescent) female patient with known Crohn's disease with severe active disease in a "colonic/Ileocolonic" distribution (Paris L2 or L3, B1) despite adequate first line immunosuppression and one or more associated risk factors for venous thrombosis.

(1-3 = inappropriate, 4-6 = uncertain, 7-9 = appropriate)

* 1. Offer no thromboprophylaxis

* 2. Offer thromboprophylaxis until discharged home

* 3. Offer thromboprophylaxis until in clinical remission

Chapter 2a: Flaring patients with Crohn's disease requiring hospital admission

K) In a 15 year old (post-pubescent) male patient with known Crohn’s disease with severe active disease in a “colonic/ileocolonic” distribution (Paris L2 or L3, B1) despite adequate first line immunosuppression and no associated risk factors for venous thrombosis.

(1-3 = inappropriate, 4-6 = uncertain, 7-9 = appropriate)

* 1. Offer no thromboprophylaxis

* 2. Offer thromboprophylaxis until discharged home

* 3. Offer thromboprophylaxis until in clinical remission

Chapter 2a: Flaring patients with Crohn's disease requiring hospital admission

L) In a 15 year old (post-pubescent) male patient with known Crohn’s disease with severe active disease in a “colonic/ileocolonic” distribution (Paris L2 or L3, B1) despite adequate first line immunosuppression and one or more associated risk factors for venous thrombosis.

(1-3 = inappropriate, 4-6 = uncertain, 7-9 = appropriate)

* 1. Offer no thromboprophylaxis

* 2. Offer thromboprophylaxis until discharged home

* 3. Offer thromboprophylaxis until in clinical remission

Thromboprophylaxis in paediatric IBD - A RAND appropriateness panel. Post-meeting questionnaire

Chapter 2b: Flaring patients with ulcerative colitis requiring hospital admission

A) In a 9 year old (pre-pubescent) patient with known ulcerative colitis with severe active disease in a “proctitis/left sided colitis” distribution (Paris E1 or E2) despite adequate first line immunosuppression and no associated risk factors for venous thrombosis.

(1-3 = inappropriate, 4-6 = uncertain, 7-9 = appropriate)

* 1. Offer no thromboprophylaxis

* 2. Offer thromboprophylaxis until discharged home

* 3. Offer thromboprophylaxis until in clinical remission

Chapter 2b: Flaring patients with ulcerative colitis requiring hospital admission

B) In a 9 year old (pre-pubescent) patient with known ulcerative colitis with severe active disease in a “proctitis/left sided colitis” distribution (Paris E1 or E2) despite adequate first line immunosuppression and one or more associated risk factors for venous thrombosis.

(1-3 = inappropriate, 4-6 = uncertain, 7-9 = appropriate)

* 1. Offer no thromboprophylaxis

* 2. Offer thromboprophylaxis until discharged home

* 3. Offer thromboprophylaxis until in clinical remission

Thromboprophylaxis in paediatric IBD - A RAND appropriateness panel. Post-meeting questionnaire

Chapter 2b: Flaring patients with ulcerative colitis requiring hospital admission

C) In a 15 year old (post-pubescent) female patient with known ulcerative colitis with severe active disease in a "proctitis/left sided colitis" distribution (Paris E1 or E2) despite adequate first line immunosuppression and no associated risk factors for venous thrombosis.

(1-3 = inappropriate, 4-6 = uncertain, 7-9 = appropriate)

* 1. Offer no thromboprophylaxis

* 2. Offer thromboprophylaxis until discharged home

* 3. Offer thromboprophylaxis until in clinical remission

Chapter 2b: Flaring patients with ulcerative colitis requiring hospital admission

D). In a 15 year old (post-pubescent) female patient with known ulcerative colitis with severe active disease in a "proctitis/left sided colitis" distribution (Paris E1 or E2) despite adequate first line immunosuppression and one or more associated risk factors for venous thrombosis.

(1-3 = inappropriate, 4-6 = uncertain, 7-9 = appropriate)

* 1. Offer no thromboprophylaxis

* 2. Offer thromboprophylaxis until discharged home

* 3. Offer thromboprophylaxis until in clinical remission

Chapter 2b: Flaring patients with ulcerative colitis requiring hospital admission

E) In a 15 year old (post-pubescent) male patient with known ulcerative colitis with severe active disease in a "proctitis/left sided colitis" distribution (Paris E1 or E2) despite adequate first line immunosuppression and no associated risk factors for venous thrombosis.

(1-3 = inappropriate, 4-6 = uncertain, 7-9 = appropriate)

* 1. Offer no thromboprophylaxis

* 2. Offer thromboprophylaxis until discharged home

* 3. Offer thromboprophylaxis until in clinical remission

Chapter 2b: Flaring patients with ulcerative colitis requiring hospital admission

F) In a 15 year old (post-pubescent) male patient with known ulcerative colitis with severe active disease in a “proctitis/left sided colitis” distribution (Paris E1 or E2) despite adequate first line immunosuppression and one or more associated risk factors for venous thrombosis.

(1-3 = inappropriate, 4-6 = uncertain, 7-9 = appropriate)

* 1. Offer no thromboprophylaxis

* 2. Offer thromboprophylaxis until discharged home

* 3. Offer thromboprophylaxis until in clinical remission

Chapter 2b: Flaring patients with ulcerative colitis requiring hospital admission

G) In a 9 year old (pre-pubescent) patient with known ulcerative colitis with severe active disease in an "extensive colitis/pancolitis" distribution (Paris E3 or E4) despite adequate first line immunosuppression and no associated risk factors for venous thrombosis.

(1-3 = inappropriate, 4-6 = uncertain, 7-9 = appropriate)

* 1. Offer no thromboprophylaxis

* 2. Offer thromboprophylaxis until discharged home

* 3. Offer thromboprophylaxis until in clinical remission

Chapter 2b: Flaring patients with ulcerative colitis requiring hospital admission

H) In a 9 year old (pre-pubescent) patient with known ulcerative colitis with severe active disease in an “extensive colitis/pancolitis” distribution (Paris E3 or E4) despite adequate first line immunosuppression and one or more associated risk factors for venous thrombosis.

(1-3 = inappropriate, 4-6 = uncertain, 7-9 = appropriate)

* 1. Offer no thromboprophylaxis

* 2. Offer thromboprophylaxis until discharged home

* 3. Offer thromboprophylaxis until in clinical remission

Thromboprophylaxis in paediatric IBD - A RAND appropriateness panel. Post-meeting questionnaire

Chapter 2b: Flaring patients with ulcerative colitis requiring hospital admission

I) In a 15 year old (post-pubescent) female patient with known ulcerative colitis with severe active disease in an "extensive colitis/pancolitis" distribution (Paris E3 or E4) despite adequate first line immunosuppression and no associated risk factors for venous thrombosis.

(1-3 = inappropriate, 4-6 = uncertain, 7-9 = appropriate)

* 1. Offer no thromboprophylaxis

* 2. Offer thromboprophylaxis until discharged home

* 3. Offer thromboprophylaxis until in clinical remission

Thromboprophylaxis in paediatric IBD - A RAND appropriateness panel. Post-meeting questionnaire

Chapter 2b: Flaring patients with ulcerative colitis requiring hospital admission

J) In a 15 year old (post-pubescent) female patient with known ulcerative colitis with severe active disease in an “extensive colitis/pancolitis” distribution (Paris E3 or E4) despite adequate first line immunosuppression and one or more associated risk factors for venous thrombosis.

(1-3 = inappropriate, 4-6 = uncertain, 7-9 = appropriate)

* 1. Offer no thromboprophylaxis

* 2. Offer thromboprophylaxis until discharged home

* 3. Offer thromboprophylaxis until in clinical remission

Chapter 2b: Flaring patients with ulcerative colitis requiring hospital admission

K) In a 15 year old (post-pubescent) male patient with known ulcerative colitis with severe active disease in an “extensive colitis/pancolitis” distribution (Paris E3 or E4) despite adequate first line immunosuppression and no associated risk factors for venous thrombosis.

(1-3 = inappropriate, 4-6 = uncertain, 7-9 = appropriate)

* 1. Offer no thromboprophylaxis

* 2. Offer thromboprophylaxis until discharged home

* 3. Offer thromboprophylaxis until in clinical remission

Thromboprophylaxis in paediatric IBD - A RAND appropriateness panel. Post-meeting questionnaire

Chapter 2b: Flaring patients with ulcerative colitis requiring hospital admission

L) In a 15 year old (post-pubescent) male patient with known ulcerative colitis with severe active disease in an "extensive colitis/pancolitis" distribution (Paris E3 or E4) despite adequate first line immunosuppression and one or more associated risk factors for venous thrombosis.

(1-3 = inappropriate, 4-6 = uncertain, 7-9 = appropriate)

* 1. Offer no thromboprophylaxis

* 2. Offer thromboprophylaxis until discharged home

* 3. Offer thromboprophylaxis until in clinical remission

Thromboprophylaxis in paediatric IBD - A RAND appropriateness panel. Post-meeting questionnaire

Chapter 4: Additional

In any patient on thromboprophylaxis:

(1-3 = inappropriate, 4-6 = uncertain, 7-9 = appropriate)

* 1. Rate the appropriateness of endoscopy with biopsies without interrupting heparin.

Thromboprophylaxis in paediatric IBD - A RAND appropriateness panel. Post-meeting questionnaire

END OF SURVEY

Thank you for completing the survey - please email: richard.hansen@ggc.scot.nhs.uk if you have any questions or comments

1. Alternatively please document your comments below.
