# Supplementary material for: Thromboprophylaxis Use in Paediatric Inflammatory Bowel Disease: An International RAND Appropriateness Panel
Source: J Crohns Colitis. 2022 May 24;16(10):1609–16. doi: 10.1093/ecco-jcc/jjac073 (PMC9624289; doi:10.1093/ecco-jcc/jjac073)
Supplement: jjac073_suppl_Supplementary_Tables [file jjac073_suppl_supplementary_tables.docx]

**Supplementary Table 1:** Paediatric gastroenterologists convened for RAND panel on thromboprophylaxis use in paediatric inflammatory bowel disease

| RAND Panellist | Affiliation |
| --- | --- |
| Franco Torrente | Department of Paediatric Gastroenterology, Addenbrooke’s Hospital, Cambridge, UK |
| Eric Benchimol | SickKids Inflammatory Bowel Disease Centre, Division of Gastroenterology, Hepatology and Nutrition, Hospital for Sick Children, Toronto, Canada. |
| Lissy de Ridder | Pediatric Gastroenterology Department, Erasmus MC Sophia Children Hospital, Rotterdam, The Netherlands. |
| Nick M. Croft | Blizard Institute, Barts and the London School of Medicine, Queen Mary University of London, London, UK. |
| Jochen Kammermeier | Department of Paediatric Gastroenterology, Evelina London Children’s Hospital, London, UK. |
| David R. Mack | CHEO IBD Centre, Children’s Hospital of Eastern Ontario Ottawa, Canada. |
| Dan Turner | Shaare Zedek Medical Centre- The Hebrew The Juliet Keidan Institute of Pediatric Gastroenterology and Nutrition, Shaare Zedek Medical Center, The Hebrew University of Jerusalem, Israel. |
| David C. Wilson | University of Edinburgh, Child Life and Health, Edinburgh, UK. |
| Javier Martín-de-Carpi | Department of Pediatric Gastroenterology, Hepatology and Nutrition, Hospital Sant Joan de Déu, Barcelona, Spain. |
| Jiri Bronsky | Paediatric Gastroenterology Unit, Department of Paediatrics, University Hospital Motol, Prague, Czech Republic. |
| Jorge Amil-Dias | Department of Paediatrics, Centro Hospitalar São João, Porto, Portugal. |
| Richard Hansen | Department of Paediatric Gastroenterology, Royal Hospital for Children, Glasgow, UK. |
| Kaija-Leena Kolho  (unable to attend meeting so withdrawn as panellist) | University of Helsinki, Helsinki, Finland. |
| Gigi Veereman  (unable to attend meeting so withdrawn as panellist) | University Hospital Brussels, Brussels, Belgium. |

**Supplementary Table 2:** Reference list provided to panel after literature review, divided into subcategories

| **Category/Brief Title** | **Reference** |
| --- | --- |
| **Guideline Documents** | |
| ACG Guideline for adult Crohn’s | (1) |
| ACG Guideline for adult ulcerative colitis | (2) |
| BSG Guideline for adult IBD patients | (3) |
| CAG consensus statements for VTE prevention in IBD patients | (4) |
| ECCO guideline for extra-intestinal manifestations in adult IBD patients | (5) |
| ESPGHAN ECCO guideline for paediatric ulcerative colitis- Part 1, ambulatory care | (6) |
| ESPGHAN ECCO guideline for paediatric ulcerative colitis- Part 2, acute severe colitis | (7) |
| ESPGHAN ECCO guideline for paediatric Crohn’s disease- update | (8) |
| **Rates of Venous Thrombosis** | |
| Aardoom & Klomberg JCC 2021 | (9) |
| Kappelman Gut 2011 | (10) |
| Kuenzig JCC 2021 | (11) |
| **Safety** | |
| Shen APT 2007 | (12) |
| Story JPGN 2021 | (13) |
| **Thrombosis After Discharge** | |
| McCurdy Dig Dis Sci 2021 | (14) |

**Supplementary Table 3:** Numerical Results and Outcomes for RAND Panel Statements

| **Statements*** | **Median** | **Disagreement index** | **Standard Deviation** | **Interpercentile range** | **Category** |
| --- | --- | --- | --- | --- | --- |
| **Section 1: New onset acute severe colitis** | | | | | |
| In a 9-year-old (pre-pubescent) patient with newly diagnosed acute severe colitis with one or more  associated risk factors for venous thrombosis after limited sigmoidoscopy | | | | | |
| Offer no thromboprophylaxis | 2.0 | 0.26 | 1.73 | 1.7 | **Inappropriate** |
| Offer thromboprophylaxis until discharged home | 8.5 | 0.13 | 1.73 | 1.0 | **Appropriate** |
| Offer thromboprophylaxis until clinical remission | 5.0 | 0.94 | 2.00 | 2.7 | **Uncertain** |
| In a 15-year-old (post-pubescent) female patient with newly diagnosed acute severe colitis with one  or more associated risk factors for venous thrombosis after limited sigmoidoscopy | | | | | |
| Offer no thromboprophylaxis | 1.5 | 0.14 | 0.97 | 1.0 | **Inappropriate** |
| Offer thromboprophylaxis until discharged home | 9.0 | 0.09 | 0.67 | 0.7 | **Appropriate** |
| Offer thromboprophylaxis until clinical remission | 6.0 | 0.50 | 1.93 | 1.4 | **Uncertain** |
| In a 15-year-old (post-pubescent) male patient with newly diagnosed acute severe colitis with one  or more associated risk factors for venous thrombosis after limited sigmoidoscopy | | | | | |
| Offer no thromboprophylaxis | 2.0 | 0.13 | 1.76 | 1.0 | **Inappropriate** |
| Offer thromboprophylaxis until discharged home | 8.5 | 0.13 | 1.73 | 1.0 | **Appropriate** |
| Offer thromboprophylaxis until clinical remission | 5.5 | 0.94 | 2.15 | 2.7 | **Uncertain** |
| **Section 2a: Flaring patients severe Crohn’s disease requiring admission, despite adequate first line immunosuppression** | | | | | |
| In a 9-year-old (pre-pubescent) patient with known Crohn’s disease with severe active disease in an  “ileal” distribution (Paris L1, B1) despite adequate first line immunosuppression and no associated  risk factors for venous thrombosis | | | | | |
| Offer no thromboprophylaxis | 4.5 | 0.97 | 2.39 | 3.0 | **Uncertain** |
| Offer thromboprophylaxis until discharged home | 5.5 | 0.77 | 2.34 | 2.4 | **Uncertain** |
| Offer thromboprophylaxis until clinical remission | 3.5 | 0.46 | 1.62 | 2.7 | **Uncertain** |
| In a 9-year-old (pre-pubescent) patient with known Crohn’s disease with severe active disease in an  “ileal” distribution (Paris L1, B1) despite adequate first line immunosuppression and one or more  associated risk factors for venous thrombosis | | | | | |
| Offer no thromboprophylaxis | 3.0 | 0.22 | 1.50 | 1.0 | **Inappropriate** |
| Offer thromboprophylaxis until discharged home | 7.0 | 0.26 | 1.82 | 1.4 | **Appropriate** |
| Offer thromboprophylaxis until clinical remission | 4.0 | 0.52 | 1.65 | 2.0 | **Uncertain** |
| In a 15-year-old (post-pubescent) female patient with known Crohn’s disease with severe active  disease in an “ileal” distribution (Paris L1, B1) despite adequate first line immunosuppression and no  associated risk factors for venous thrombosis. | | | | | |
| Offer no thromboprophylaxis | 4.5 | 1.45† | 2.49 | 3.4 | **Uncertain** |
| Offer thromboprophylaxis until discharged home | 5.5 | 1.44† | 2.47 | 3.7 | **Uncertain** |
| Offer thromboprophylaxis until clinical remission | 3.5 | 0.75 | 2.23 | 4.0 | **Uncertain** |
| In a 15-year-old (post-pubescent) female patient with known Crohn’s disease with severe active  disease in an “ileal” distribution (Paris L1, B1) despite adequate first line immunosuppression and one  or more associated risk factors for venous thrombosis. | | | | | |
| Offer no thromboprophylaxis | 2.5 | 0.30 | 1.88 | 1.7 | **Inappropriate** |
| Offer thromboprophylaxis until discharged home | 7.5 | 0.16 | 1.92 | 1.0 | **Appropriate** |
| Offer thromboprophylaxis until clinical remission | 4.5 | 0.47 | 1.64 | 1.7 | **Uncertain** |
| In a 15-year-old (post-pubescent) male patient with known Crohn’s disease with severe active  disease in an “ileal” distribution (Paris L1, B1) despite adequate first line immunosuppression and no  associated risk factors for venous thrombosis. | | | | | |
| Offer no thromboprophylaxis | 4.5 | 1.44† | 2.53 | 3.7 | **Uncertain** |
| Offer thromboprophylaxis until discharged home | 5.5 | 0.97 | 2.41 | 3.0 | **Uncertain** |
| Offer thromboprophylaxis until clinical remission | 3.5 | 0.64 | 2.01 | 3.4 | **Uncertain** |
| In a 15-year-old (post-pubescent) male patient with known Crohn’s disease with severe active  disease in an “ileal” distribution (Paris L1, B1) despite adequate first line immunosuppression and one  or more associated risk factors for venous thrombosis. | | | | | |
| Offer no thromboprophylaxis | 2.5 | 0.37 | 0.37 | 2.0 | **Inappropriate** |
| Offer thromboprophylaxis until discharged home | 7.5 | 0.30 | 1.95 | 1.7 | **Appropriate** |
| Offer thromboprophylaxis until clinical remission | 4.5 | 0.52 | 1.76 | 2.0 | **Uncertain** |
| In a 9-year-old (pre-pubescent) patient with known Crohn’s disease with severe active disease in a  “colonic/ileocolonic” distribution (Paris L2 or L3, B1) despite adequate first line immunosuppression  and no associated risk factors for venous thrombosis. | | | | | |
| Offer no thromboprophylaxis | 4.0 | 1.44† | 2.13 | 3.7 | **Uncertain** |
| Offer thromboprophylaxis until discharged home | 7.0 | 0.68 | 2.04 | 2.4 | **Appropriate** |
| Offer thromboprophylaxis until clinical remission | 4.0 | 0.62 | 1.93 | 2.7 | **Uncertain** |
| In a 9-year-old (pre-pubescent) patient with known Crohn’s disease with severe active disease in a  “colonic/ileocolonic” distribution (Paris L2 or L3, B1) despite adequate first line immunosuppression  and one or more associated risk factors for venous thrombosis. | | | | | |
| Offer no thromboprophylaxis | 3.0 | 0.46 | 1.73 | 2.7 | **Inappropriate** |
| Offer thromboprophylaxis until discharged home | 8.0 | 0.26 | 1.51 | 1.7 | **Appropriate** |
| Offer thromboprophylaxis until clinical remission | 4.5 | 0.47 | 1.65 | 1.7 | **Uncertain** |
| In a 15-year-old (post-pubescent) female patient with known Crohn’s disease with severe active  disease in a “colonic/Ileocolonic” distribution (Paris L2 or L3, B1) despite adequate first line  immunosuppression and no associated risk factors for venous thrombosis. | | | | | |
| Offer no thromboprophylaxis | 3.5 | 0.76 | 2.54 | 3.1 | **Uncertain** |
| Offer thromboprophylaxis until discharged home | 7.0 | 0.26 | 2.15 | 1.4 | **Appropriate** |
| Offer thromboprophylaxis until clinical remission | 4.5 | 0.81 | 1.95 | 2.7 | **Uncertain** |
| In a 15-year-old (post-pubescent) female patient with known Crohn’s disease with severe active  disease in a “colonic/Ileocolonic” distribution (Paris L2 or L3, B1) despite adequate first line  immunosuppression and one or more associated risk factors for venous thrombosis. | | | | | |
| Offer no thromboprophylaxis | 2.5 | 0.26 | 1.38 | 1.7 | **Inappropriate** |
| Offer thromboprophylaxis until discharged home | 8.0 | 0.29 | 1.36 | 2.0 | **Appropriate** |
| Offer thromboprophylaxis until clinical remission | 4.5 | 0.97 | 1.82 | 3.0 | **Uncertain** |
| In a 15-year-old (post-pubescent) male patient with known Crohn’s disease with severe active  disease in a “colonic/ileocolonic” distribution (Paris L2 or L3, B1) despite adequate first line  immunosuppression and no associated risk factors for venous thrombosis. | | | | | |
| Offer no thromboprophylaxis | 3.5 | 1.02† | 2.38 | 3.1 | **Uncertain** |
| Offer thromboprophylaxis until discharged home | 7.0 | 0.42 | 2.13 | 1.7 | **Appropriate** |
| Offer thromboprophylaxis until clinical remission | 4.5 | 0.62 | 2.05 | 2.7 | **Uncertain** |
| In a 15-year-old (post-pubescent) male patient with known Crohn’s disease with severe active  disease in a “colonic/ileocolonic” distribution (Paris L2 or L3, B1) despite adequate first line  immunosuppression and one or more associated risk factors for venous thrombosis. | | | | | |
| Offer no thromboprophylaxis | 2.0 | 0.26 | 1.30 | 1.7 | **Inappropriate** |
| Offer thromboprophylaxis until discharged home | 8.0 | 0.24 | 1.34 | 1.7 | **Appropriate** |
| Offer thromboprophylaxis until clinical remission | 4.5 | 0.94 | 1.78 | 2.7 | **Uncertain** |
| **Section 2b: Flaring patients with severe ulcerative colitis requiring hospital admission, despite adequate first line immunosuppression** | | | | | |
| In a 9-year-old (pre-pubescent) patient with known ulcerative colitis with severe active disease in a  “proctitis/left sided colitis” distribution (Paris E1 or E2) despite adequate first line  immunosuppression and no associated risk factors for venous thrombosis. | | | | | |
| Offer no thromboprophylaxis | 3.5 | 0.56 | 2.76 | 2.7 | **Uncertain** |
| Offer thromboprophylaxis until discharged home | 7.0 | 0.42 | 2.41 | 1.7 | **Appropriate** |
| Offer thromboprophylaxis until clinical remission | 4.5 | 0.62 | 1.89 | 1.0 | **Uncertain** |
| In a 9-year-old (pre-pubescent) patient with known ulcerative colitis with severe active disease in a  “proctitis/left sided colitis” distribution (Paris E1 or E2) despite adequate first line  immunosuppression and one or more associated risk factors for venous thrombosis. | | | | | |
| Offer no thromboprophylaxis | 2.0 | 0.16 | 1.76 | 1.0 | **Inappropriate** |
| Offer thromboprophylaxis until discharged home | 8.0 | 0.11 | 0.41 | 0.7 | **Appropriate** |
| Offer thromboprophylaxis until clinical remission | 4.5 | 0.52 | 1.60 | 2.0 | **Uncertain** |
| In a 15-year-old (post-pubescent) female patient with known ulcerative colitis with severe active  disease in a “proctitis/left sided colitis” distribution (Paris E1 or E2) despite adequate first line  immunosuppression and no associated risk factors for venous thrombosis. | | | | | |
| Offer no thromboprophylaxis | 3.0 | 0.48 | 1.68 | 2.4 | **Inappropriate** |
| Offer thromboprophylaxis until discharged home | 7.0 | 0.48 | 2.53 | 2.4 | **Appropriate** |
| Offer thromboprophylaxis until clinical remission | 4.5 | 0.62 | 1.97 | 2.7 | **Uncertain** |
| In a 15-year-old (post-pubescent) female patient with known ulcerative colitis with severe active  disease in a “proctitis/left sided colitis” distribution (Paris E1 or E2) despite adequate first line  immunosuppression and one or more associated risk factors for venous thrombosis. | | | | | |
| Offer no thromboprophylaxis | 1.0 | 0.13 | 1.56 | 1.0 | **Inappropriate** |
| Offer thromboprophylaxis until discharged home | 8.0 | 0.24 | 1.54 | 1.7 | **Appropriate** |
| Offer thromboprophylaxis until clinical remission | 4.5 | 0.97 | 0.97 | 3.0 | **Uncertain** |
| In a 15-year-old (post-pubescent) male patient with known ulcerative colitis with severe active  disease in a “proctitis/left sided colitis” distribution (Paris E1 or E2) despite adequate first line  immunosuppression and no associated risk factors for venous thrombosis. | | | | | |
| Offer no thromboprophylaxis | 3.0 | 0.56 | 2.66 | 2.7 | **Inappropriate** |
| Offer thromboprophylaxis until discharged home | 7.0 | 0.56 | 2.57 | 2.7 | **Appropriate** |
| Offer thromboprophylaxis until clinical remission | 4.0 | 0.65 | 1.93 | 3.0 | **Uncertain** |
| In a 15-year-old (post-pubescent) male patient with known ulcerative colitis with severe active  disease in a “proctitis/left sided colitis” distribution (Paris E1 or E2) despite adequate first line  immunosuppression and one or more associated risk factors for venous thrombosis. | | | | | |
| Offer no thromboprophylaxis | 1.5 | 0.13 | 1.54 | 1.0 | **Inappropriate** |
| Offer thromboprophylaxis until discharged home | 8.0 | 0.13 | 1.53 | 1.0 | **Appropriate** |
| Offer thromboprophylaxis until clinical remission | 4.5 | 0.94 | 1.88 | 2.7 | **Uncertain** |
| In a 9-year-old (pre-pubescent) patient with known ulcerative colitis with severe active disease in an  “extensive colitis/pancolitis” distribution (Paris E3 or E4) despite adequate first line  immunosuppression and no associated risk factors for venous thrombosis. | | | | | |
| Offer no thromboprophylaxis | 2.5 | 0.46 | 2.42 | 2.7 | **Inappropriate** |
| Offer thromboprophylaxis until discharged home | 7.5 | 0.16 | 2.27 | 1.0 | **Appropriate** |
| Offer thromboprophylaxis until clinical remission | 5.0 | 0.47 | 2.00 | 1.7 | **Uncertain** |
| In a 9-year-old (pre-pubescent) patient with known ulcerative colitis with severe active disease in an  “extensive colitis/pancolitis” distribution (Paris E3 or E4) despite adequate first line  immunosuppression and one or more associated risk factors for venous thrombosis. | | | | | |
| Offer no thromboprophylaxis | 1.5 | 0.24 | 1.08 | 1.7 | **Inappropriate** |
| Offer thromboprophylaxis until discharged home | 8.5 | 0.13 | 1.03 | 1.0 | **Appropriate** |
| Offer thromboprophylaxis until clinical remission | 5.5 | 0.32 | 1.76 | 1.0 | **Uncertain** |
| In a 15-year-old (post-pubescent) female patient with known ulcerative colitis with severe active  disease in an “extensive colitis/pancolitis” distribution (Paris E3 or E4) despite adequate first line  immunosuppression and no associated risk factors for venous thrombosis. | | | | | |
| Offer no thromboprophylaxis | 1.5 | 0.43 | 2.48 | 2.7 | **Inappropriate** |
| Offer thromboprophylaxis until discharged home | 8.0 | 0.20 | 2.37 | 1.4 | **Appropriate** |
| Offer thromboprophylaxis until clinical remission | 5.0 | 0.47 | 2.00 | 1.7 | **Uncertain** |
| In a 15-year-old (post-pubescent) female patient with known ulcerative colitis with severe active  disease in an “extensive colitis/pancolitis” distribution (Paris E3 or E4) despite adequate first line  immunosuppression and one or more associated risk factors for venous thrombosis. | | | | | |
| Offer no thromboprophylaxis | 1.0 | 0.13 | 0.67 | 1.0 | **Inappropriate** |
| Offer thromboprophylaxis until discharged home | 9.0 | 0.09 | 0.67 | 0.7 | **Appropriate** |
| Offer thromboprophylaxis until clinical remission | 5.5 | 0.66 | 1.86 | 1.7 | **Uncertain** |
| In a 15-year-old (post-pubescent) male patient with known ulcerative colitis with severe active  disease in an “extensive colitis/pancolitis” distribution (Paris E3 or E4) despite adequate first line  immunosuppression and no associated risk factors for venous thrombosis. | | | | | |
| Offer no thromboprophylaxis | 2.0 | 0.66 | 2.48 | 3.7 | **Inappropriate** |
| Offer thromboprophylaxis until discharged home | 8.0 | 0.26 | 2.17 | 1.7 | **Appropriate** |
| Offer thromboprophylaxis until clinical remission | 4.5 | 0.47 | 1.98 | 1.7 | **Uncertain** |
| In a 15-year-old (post-pubescent) male patient with known ulcerative colitis with severe active  disease in an “extensive colitis/pancolitis” distribution (Paris E3 or E4) despite adequate first line  immunosuppression and one or more associated risk factors for venous thrombosis. | | | | | |
| Offer no thromboprophylaxis | 1.5 | 0.13 | 0.78 | 1.0 | **Inappropriate** |
| Offer thromboprophylaxis until discharged home | 9.0 | 0.13 | 0.79 | 1.0 | **Appropriate** |
| Offer thromboprophylaxis until clinical remission | 5.5 | 0.32 | 1.85 | 1.0 | **Uncertain** |
| **Section 4: Endoscopy and thromboprophylaxis** | | | | | |
| In any patient on thromboprophylaxis, it is appropriate to perform endoscopy with biopsies without interrupting heparin | 6.0 | 0.22 | 1.54 | 1.0 | **Uncertain** |

*Panellists were asked to rate the appropriateness of the statements listed, where 1-3 = inappropriate, 4-6 = uncertain and 7-9 = appropriate.

†Denotes disagreement

E1/E2/E3/E4 refer to the Paris classification of disease extent in ulcerative colitis – respectively: proctitis, left sided disease to the splenic flexure, proximal to the splenic flexure but distal to the hepatic flexure, pancolitis; L1/L2/3 refer to the Paris classification for the distribution of disease in Crohn’s disease – respectively: distal ileal/ileocaecal disease, colonic and ileocolonic disease; B1 refers to non-stricturing, non-penetrating phenotype of Crohn’s disease

**References:**

1. Lichtenstein GR, Loftus E v., Isaacs KL, Regueiro MD, Gerson LB, Sands BE. ACG Clinical Guideline: Management of Crohn’s Disease in Adults. American Journal of Gastroenterology. 2018;113(4):481–517.

2. Rubin DT, Ananthakrishnan AN, Siegel CA, Sauer BG, Long MD. ACG Clinical Guideline: Ulcerative Colitis in Adults. American Journal of Gastroenterology. 2019;114(3):384–413.

3. Lamb CA, Kennedy NA, Raine T, Hendy PA, Smith PJ, Limdi JK, et al. British Society of Gastroenterology consensus guidelines on the management of inflammatory bowel disease in adults. Gut. 2019;68(Suppl 3):S1–106.

4. Nguyen GC, Bernstein CN, Bitton A, Chan AK, Griffiths AM, Leontiadis GI, et al. Consensus statements on the risk, prevention, and treatment of venous thromboembolism in inflammatory bowel disease: Canadian association of gastroenterology. Gastroenterology. 2014;146(3):835–48.

5. Harbord M, Annese V, Vavricka SR, Allez M, Acosta MB de, Boberg KM, et al. The first european evidence-based consensus on extra-intestinal manifestations in inflammatory bowel disease. Journal of Crohn’s and Colitis. 2016 Mar 1;10(3):239–54.

6. Turner D, Ruemmele FM, Orlanski-Meyer E, Griffiths AM, de Carpi JM, Bronsky J, et al. Management of paediatric ulcerative colitis, part 1: Ambulatory Care-An Evidence-based Guideline From European Crohn’s and Colitis Organization and European Society of Paediatric Gastroenterology, Hepatology and Nutrition. Journal of Pediatric Gastroenterology and Nutrition. 2018;67(2):257–91.

7. Turner D, Ruemmele FM, Orlanski-Meyer E, Griffiths AM, de Carpi JM, Bronsky J, et al. Management of paediatric ulcerative colitis, part 2: Acute severe colitis - An evidence-based consensus guideline from the european Crohn’s and colitis organization and the european society of paediatric gastroenterology, hepatology and nutrition. Journal of Pediatric Gastroenterology and Nutrition. 2018;67(2):292–310.

8. van Rheenen PF, Aloi M, Assa A, Bronsky J, Escher JC, Fagerberg UL, et al. The Medical Management of Paediatric Crohn’s Disease: An ECCO-ESPGHAN Guideline Update. Journal of Crohn’s and Colitis. 2021;15(2):171–94.

9. Klomberg RC, Aardoom MA, Kemos P, Ruemmele FM, van Ommen H, Croft NM, et al. The incidence and characteristics of venous thromboembolisms in paediatric-onset inflammatory bowel disease; a prospective international cohort study based on the PIBD-SETQuality Safety Registry. Journal of Crohn’s and Colitis [Internet]. 2022: in press.

10. Kappelman MD, Horvath-Puho E, Sandler RS, Rubin DT, Ullman TA, Pedersen L, et al. Thromboembolic risk among Danish children and adults with inflammatory bowel diseases: A population-based nationwide study. Gut. 2011;60(7):937–43.

11. Kuenzig ME, Bitton A, Carroll MW, Kaplan GG, Otley AR, Singh H, et al. Inflammatory Bowel Disease Increases the Risk of Venous Thromboembolism in Children: A Population-Based Matched Cohort Study. Journal of Crohn’s and Colitis. 2021;15(12):2031–40.

12. Shen J, Ran ZH, Tong JL, Xiao SD. Meta-analysis: The utility and safety of heparin in the treatment of active ulcerative colitis. Alimentary Pharmacology and Therapeutics. 2007;26(5):653–63.

13. Story E, Bijelic V, Penney C, Benchimol EI, Halton J, Mack DR. Safety of Venous Thromboprophylaxis With Low-molecular-weight Heparin in Children With Ulcerative Colitis. Journal of pediatric gastroenterology and nutrition. 2021;73(5):604–9.

14. McCurdy JD, Ellen Kuenzig M, Spruin S, Fung OW, Mallik R, Williams L, et al. Surgery and the Subtype of Inflammatory Bowel Disease Impact the Risk of Venous Thromboembolism After Hospital Discharge. Digestive Diseases and Sciences. 2022: in press.
